# Letrozole Cotreatment Reduces Unexpectedly Poor Responses in Ovarian Stimulation With Follitropin Delta: A Strategy to Prevent High Anti-Müllerian Hormone (AMH) but Poor Response

**DOI:** 10.7759/cureus.78513

**Published:** 2025-02-04

**Authors:** Hiromasa Kuroda, Kana Inukai, Akiko Shibaike, Kanako Ishii, Koichiro Lee, Masayo Yamada, Naoko Murakami, Mariko Shindo, Mika Koyama, Atsushi Haruki

**Affiliations:** 1 Obstetrics and Gynecology, Haruki Ladies Clinic, Osaka, JPN

**Keywords:** anti-müllerian hormone (amh), aromatase inhibitor, assisted reproductive technology (art), follitropin delta, infertility, letrozole, oocyte retrieval, ovarian response, ovarian stimulation, poor response

## Abstract

Background: Follitropin delta is a novel recombinant follicle-stimulating hormone preparation used for in vitro fertilization/intracytoplasmic sperm injection (IVF/ICSI). The dosage is determined using an original algorithm designed to achieve a target retrieval of 8-14 oocytes based on body weight and anti-Müllerian hormone (AMH) levels. However, unexpected poor responses, characterized by low oocyte retrieval numbers, occasionally occur in patients with high AMH levels who are otherwise expected to respond well. This study investigated whether cotreatment with letrozole reduces such poor responses.

Methods: A retrospective cohort study including 153 controlled ovarian stimulation (COS) cycles for IVF/ICSI using follitropin delta was performed at Haruki Ladies Clinic in Japan from October 2021 to March 2023. In total, 42 cycles were performed in the letrozole cotreatment group, and 111 cycles were performed in the group treated with follitropin delta alone. According to the concept of follitropin delta, seven or fewer oocytes retrieved were defined as a poor response.

Results: An unexpectedly poor response was observed at 6.0-6.9 µg daily doses of follitropin delta. The poor response was less frequent in the cotreatment group: one of 36 cycles (2.8%) in the cotreatment group and nine of 49 cycles (18.4%) in the follitropin delta alone group (p < 0.05). At daily doses of 7.0-11.9 μg, poor response was not often observed in both groups (0% vs. 3.6%). At 12.0 μg of daily dose, poor response frequently occurred in both groups. The duration of ovarian stimulation was decreased by cotreatment with letrozole (10.4 days vs. 8.7 days, p < 0.01). Letrozole cotreatment also reduced the total dosage of follitropin delta (65.2 µg vs. 53.3 µg, p < 0.01).

Conclusions: Cotreatment with letrozole may reduce unanticipated suboptimal responses in patients expected to have good responses. Additionally, it may shorten the duration of ovarian stimulation and decrease the total dosage of follitropin delta required.

## Introduction

Follitropin delta, a novel injectable used for controlled ovarian stimulation (COS) in assisted reproductive technology (ART), has been utilized globally since its initial approval in Europe in December 2016. It is the world’s first recombinant follicle-stimulating hormone (FSH) product derived from a human cell line (human embryonic retinal cells). A unique algorithm, based on body weight and serum anti-Müllerian hormone (AMH) levels, automatically determines the appropriate fixed daily dose for each individual patient [1,2].

The efficacy and safety of follitropin delta in gonadotropin-releasing hormone (GnRH) antagonist protocols have been demonstrated in randomized controlled trials (RCTs) [3-5]. A distinctive feature of follitropin delta is its unique dosing algorithm, designed to minimize the risks of hypo- and hyper-ovarian responses while optimizing the retrieval of 8-14 oocytes. Previous studies have shown that this individualized dosing algorithm reduces the risk of ovarian hyperstimulation syndrome (OHSS) without compromising pregnancy outcomes, compared to conventional dosing protocols using follitropin alfa and beta [1,3-11]. The individualized dosing for follitropin delta is increasingly being adopted as an alternative to traditional FSH dosing protocols.

Bachmann et al. conducted a retrospective study in Germany across eight institutions between 2018 and 2019, analyzing 360 COS cycles with follitropin delta. Among cases where an optimal or greater number of oocytes were expected to be retrieved, and serum AMH levels were 35 pmol/L or higher, indicating a higher risk of ovarian hyperstimulation syndrome (OHSS); 8.4% of these cases resulted in the retrieval of only ≤4 oocytes. This percentage is unexpectedly higher than the 5.7% observed in normal responders, whose serum AMH levels ranged from 15 to 35 pmol/L [12]. These findings suggest that the dose-setting algorithm for follitropin delta may still be suboptimal for high responders.

Letrozole, a third-generation aromatase inhibitor, reduces estradiol levels by inhibiting the aromatase enzyme responsible for converting androgens to estradiol [13]. This reduction triggers a negative feedback mechanism, promoting follicle development by increasing endogenous FSH secretion from the pituitary gland [14]. Letrozole is also used for COS in IVF/ICSI and is known to reduce the exogenous FSH dosage required before oocyte retrieval [15]. Several studies have shown that letrozole cotreatment in COS leads to increased oocyte retrieval, underscoring its practical benefits in clinical settings [16-19].

Based on these mechanisms and previous studies, it is hypothesized that cotreatment with letrozole can reduce unexpected poor responses in COS using follitropin delta. This study aims to evaluate whether combining letrozole with follitropin delta can mitigate poor responses and prevent unexpectedly low oocyte retrieval in patients with high AMH levels.

## Materials and methods

Study design and participants

This study was a retrospective cohort analysis of ovarian stimulation cycles using follitropin delta (Rekovelle®, USA, Ferring Pharmaceuticals, 802-24151-9, 802-24152-6, 802-24153-3) conducted at Haruki Ladies Clinic, Osaka, Japan. The study protocol was approved by the ethics review board of Haruki Ladies Clinic, and all participants provided written informed consent for the use of their clinical data.

The inclusion criteria were as follows: (1) women diagnosed as infertile without additional complications, (2) women aged between 18 and 43 years at the time of oocyte retrieval, (3) patients who had never undergone ART before, and (4) oocyte retrieval performed by a skilled physician at our facility. The exclusion criteria were as follows: (1) the presence of ovarian cysts that made oocyte puncture difficult, (2) initiation of injections outside of days 2-4 of the menstrual cycle, and (3) presence of uterine fibroids (myoma uteri) that interfered with oocyte puncture.

The patient recruitment for the study commenced on September 2021 and concluded on February 2023. A total of 153 East Asian women who underwent oocyte retrieval at the clinic between October 2021 and March 2023 were included. Data were extracted from patient medical records.

Study protocol

COS was performed using a GnRH antagonist protocol. On days 2-4 of the menstrual cycle, transvaginal ultrasonography was used to confirm the absence of follicular development, after which follitropin delta was administered. The daily dose was determined using the algorithm based on serum AMH levels measured within six months and body weight recorded within four weeks prior to injection. Daily doses ranged from 6.0 µg to 12.0 µg and remained fixed throughout the stimulation period without exception.

Letrozole (Switzerland, Novartis International AG, 21800AMY10006000) administration was at the discretion of the attending physician. In the letrozole cotreatment group, 2.5 mg of letrozole was orally administered daily for five days starting from the day follitropin delta was administered. In the monotherapy group, no additional agents, such as letrozole or clomiphene citrate, were combined with follitropin delta.

In total, 42 cycles were performed in the letrozole cotreatment group, and 111 cycles were performed in the monotherapy group. The follitropin delta dose was administered subcutaneously by self-injection, without adjustment based on follicular development. Follicle development was monitored using transvaginal ultrasonography, measuring the follicles’ oblique diameters. When the largest follicle reached an oblique diameter of 14 mm, patients began daily subcutaneous self-injections of the GnRH antagonist cetrorelix acetate (Cetrotide®, Merck Serono S.A., Darmstadt, Germany) at a dose of 0.25 mg.

Oocyte retrieval was scheduled when the largest follicle reached an oblique diameter of ≥ 17 mm. Approximately 38 h before retrieval, patients were administered 250 µg of choriogonadotropin alfa (Ovidrel®, Merck Serono S.A.) subcutaneously and/or 300 µg of buserelin acetate (buserelin nasal solution 0.15% [F], Fuji Pharma Co., Japan) intranasally twice. Oocyte retrieval was performed transvaginally under intravenous or local anesthesia, and IVF or ICSI procedures were subsequently carried out.

Evaluation of indicators

A poor response was defined as the retrieval of fewer than eight oocytes, based on clinical trial data for follitropin delta indicating that the optimal oocyte retrieval range is 8-14. Therefore, those with less than eight oocytes retrieved were classified as the poor response group, and those with eight or more oocytes retrieved were classified as the good response group [3]. Age, weight, body mass index (BMI), duration of infertility, history of pregnancy and miscarriage, and serum AMH levels were compared between poor and good responders. The number of oocytes retrieved, percentage of poor responders, duration of follitropin delta treatment, and total dosage of follitropin delta were compared between the letrozole cotreatment and monotherapy groups.

Statistical analysis

The Shapiro-Wilk test was used to assess the normality of data distribution. Normally distributed variables are expressed as means ± standard deviation, while non-normally distributed variables are presented as medians (minimum-maximum). Fisher’s exact test was used for nominal variables, the Mann-Whitney U test for non-normally distributed quantitative variables, Student’s t-test for normally distributed variables with equal variance, and Welch’s t-test for those with unequal variance.

The significance level was set at p < 0.05. Odds ratios and 95% confidence intervals (CIs) were calculated using multivariate logistic regression analysis, adjusting for letrozole administration, age, AMH levels, weight, and BMI. Statistical analyses were performed using R software (R Development Core Team, Austria).

## Results

Patient characteristics

Table 1 presents the patients’ background and reproductive characteristics. Since none of the variables followed a normal distribution, they were expressed as medians and were analyzed using the Mann-Whitney U test. The follitropin delta alone and letrozole cotreatment groups showed no significant differences in age, serum AMH levels, body weight, BMI, duration of infertility, number of pregnancies, or number of abortions.

**Table 1:** Background and reproductive characteristics of women undergoing ovarian stimulation with follitropin delta with or without letrozole cotreatment All variables were non-normally distributed and analyzed using the Mann–Whitney U test. The follitropin delta alone group and the letrozole cotreatment group showed no significant differences in age, serum AMH level, body weight, BMI, infertile period, number of pregnancies, and number of abortions. AMH: anti-Müllerian hormone, BMI: body mass index

Characteristics	Follitropin delta alone (n = 42)			Letrozole cotreatment (n = 111)			z-value	p-value
	Median	Minimum	Maximum	Median	Minimum	Maximum		
Age (years)	33	28	43	32	25	41	1.84	0.07
Serum AMH (ng/mL)	6.52	3.96	16.54	7.21	2.95	23.81	0.54	0.59
Body weight (kg)	50.3	41.0	61.0	50.9	41.0	61.5	0.69	0.49
BMI (kg/m^2^)	19.4	15.8	24.7	19.9	16.0	23.7	0.84	0.40
Duration of infertility (months)	22	10	45	23	11	42	0.69	0.49
No. of pregnancies	1	0	3	0	0	2	1.47	0.14
No. of abortions	0	0	2	0	0	2	0.15	0.88

Oocyte retrieval distribution

**Figure 1:**
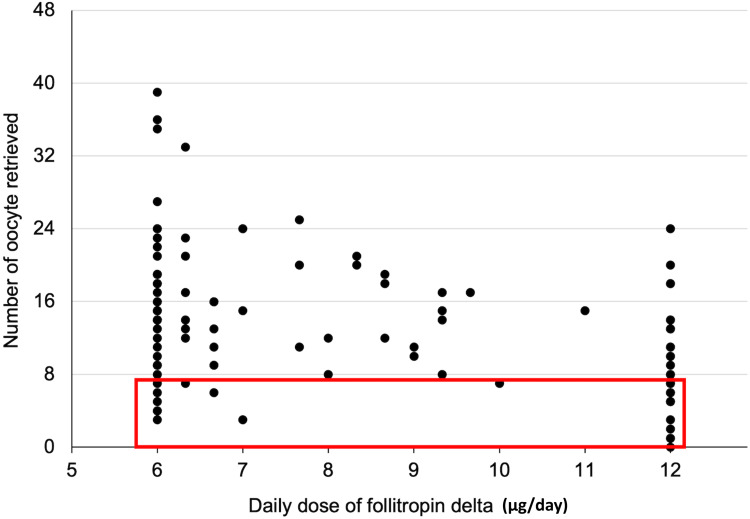
Scatterplot showing the number of oocytes retrieved for each daily dose of follitropin delta The red box highlights cycles with fewer than eight oocytes retrieved. Poor responses occurred frequently in patients with 12.0 µg/day of a follitropin delta dose who had low serum AMH levels. As not expected, poor responses happened also in patients with 6.0–6.9 µg/day of follitropin delta doses who had high serum AMH levels. Poor responses less commonly occurred in the 7.0–11.9 µg/day patient group.

The dot plot shown in Figure 1 illustrates the distribution of the number of oocytes retrieved across different follitropin delta doses. The red box highlights cycles with fewer than eight oocytes retrieved, categorized as poor responses. Poor responses were frequently observed in patients with low serum AMH levels receiving 12.0 µg/day of follitropin delta, as expected. Unexpectedly, poor responses were also identified in patients with high serum AMH levels receiving 6.0-6.9 µg/day of follitropin delta, despite typically being considered high responders. Conversely, poor responses were less common in patients receiving 7.0-11.9 µg/day, which is the expected normal response range, compared to those receiving 6.0-6.9 µg/day.

Poor response rates with letrozole cotreatment

Table 2 presents the poor response rates for each follitropin delta dose, comparing outcomes with and without letrozole cotreatment. Across the total study population, the poor response rate was significantly higher in the follitropin delta alone group (22.5%; 25 out of 111 cycles) compared to the letrozole cotreatment group (7.1%; 3 out of 42 cycles) (p < 0.05).

**Table 2:** Poor response rates in ovarian stimulation using follitropin delta with or without letrozole cotreatment Poor response rate is the proportion of fewer than eight oocytes retrieved. Patients in the follitropin delta alone group were administered only with follitropin delta and those in the letrozole cotreatment group with follitropin delta and letrozole. Analyzed by Fisher’s exact test.

Daily dose of follitropin delta (μg)	Follitropin delta alone			Letrozole cotreatment			p-value
	No. of poor responses	No. of cycles	Poor response rates (%)	No. of poor responses	No. of cycles	Poor response rates (%)	
6.0–6.9	9	49	18.4	1	36	2.8	<0.05
7.0–11.9	1	28	3.6	0	3	0	1
12.0	15	34	66.7	2	3	44.1	0.58
Total	25	111	22.5	3	42	7.1	<0.05

Subgroup analysis based on the follitropin delta dose revealed additional patterns. Among patients receiving 6.0-6.9 µg/day, where high AMH levels generally predict a good response, the poor response rate was 18.4% (9 of 49 cycles) in the follitropin delta alone group but was significantly lower at 2.8% (1 of 36 cycles) in the letrozole cotreatment group (p < 0.05). For patients receiving 7.0-11.9 µg/day, poor responses were rare in both groups. The poor response rate was 3.6% (1 of 28 cycles) in the follitropin delta alone group, while no poor responses were observed in the letrozole cotreatment group (0 of 3 cycles), although the difference was not significant (p = 1.00). Among patients receiving 12.0 µg/day of follitropin delta, who typically have low serum AMH levels, the poor response rate was 44.1% (15 of 34 cycles) in the follitropin delta alone group and 66.7% (two of 3 cycles) in the letrozole cotreatment group. This difference was not significant (p = 0.58), although the small number of cases in the letrozole cotreatment group likely reduced the statistical power.

Logistic regression analysis

A multivariate logistic regression analysis was performed to further evaluate the effect of letrozole cotreatment on the risk of poor response in patients receiving 6.0-6.9 µg/day of follitropin delta (Table 3). After adjusting for age, serum AMH levels, body weight, and BMI, the adjusted odds ratio for poor response in the letrozole cotreatment group was 0.12 (95% CI: 0.02-0.61, p < 0.05). This finding indicates that letrozole significantly reduced the risk of hyporesponsiveness in this subgroup.

**Table 3:** Logistic regression analysis evaluating the impact of letrozole cotreatment vs. follitropin delta alone on the risk of poor response AMH: anti-Müllerian hormone, BMI: body mass index, CI: confidence interval, SE: standard error

Variables	Univariable associations with poor response					Multivariable associations with poor response				
	Crude odds ratio	95% Cl	β	SE	p-value	Crude odds ratio	95% Cl	β	SE	p-value
Letrozole cotreatment	0.10	0.02 to 0.48	-2.27	0.79	<0.01	0.12	0.02 to 0.61	-2.11	0.83	<0.05
Age	1.03	0.89 to 1.19	0.03	0.07	0.71	1.02	0.87 to 1.20	0.02	0.08	0.77
Serum AMH (ng/mL)	0.91	0.74 to 1.11	-0.10	0.11	0.35	0.96	0.78 to 1.17	-0.05	0.10	0.66
Body weight (kg)	1.01	0.93 to 1.10	0.01	0.04	0.81	0.86	1.04 to 1.24	0.04	0.09	0.70
BMI (kg/m^2^)	1.01	0.80 to 1.26	0.01	0.11	0.96	0.89	0.56 to 1.42	-0.11	0.24	0.63

Duration and dosage of ovarian stimulation

Table 4 compares the duration of ovarian stimulation and the total dosage of follitropin delta administered with and without letrozole cotreatment. The addition of letrozole significantly reduced the duration of follitropin delta treatment (10.4 ± 1.1 vs. 8.7 ± 2.2 days, p < 0.01) and decreased the total dose of follitropin delta required before oocyte retrieval (65.2 ± 7.0 vs. 53.3 ± 15.0 µg, p < 0.01).

**Table 4:** Duration of ovarian stimulation and total dosage of follitropin delta administered with or without letrozole cotreatment SD: standard deviation

Follitropin delta	Follitropin delta alone		Letrozole cotreatment		t-value	p-value
	Mean	SD	Mean	SD		
Duration of ovarian stimulation (days)	10.4	1.1	8.7	2.2	6.18	< 0.01
Total dosage of follitropin delta (µg)	65.2	7.0	53.3	15.0	6.58	< 0.01

Adverse events

Adverse events were limited to OHSS. Moderate or severe OHSS occurred in two cases in the follitropin delta alone group, while no cases of OHSS were observed in the letrozole cotreatment group (p = 0.16).

## Discussion

COS with follitropin delta is designed to achieve an optimal oocyte retrieval range of 8-14, using a unique dosing algorithm based on AMH levels and body weight. However, we observed unexpected poor responses in high-AMH patients receiving 6.0-6.9 µg/day, despite these doses typically being associated with good ovarian responses. This study demonstrated that letrozole cotreatment can effectively reduce such unexpected poor responses without increasing the risk of OHSS.

No studies previously have investigated the impact of letrozole cotreatment on the number of oocytes retrieved in COS using follitropin delta. Bülow reported an RCT examining the other gonadotropin preparations, which compared clinical outcomes in 159 cycles with and without letrozole cotreatment [19]. This study found no increase in the number of oocytes retrieved with letrozole cotreatment. The study population consisted exclusively of normal responders with serum AMH levels of 8-32 pmol/L, excluding the high AMH cases in which our study observed an increase in the number of oocytes retrieved with letrozole cotreatment. Therefore, it is not possible to directly compare the high AMH group receiving 6.0-6.9 µg/day of follitropin delta in our study with the population in Bülow’s RCT. However, our study similarly found that among normal-to-poor responders receiving 7.0-12.0 µg/day of follitropin delta, letrozole cotreatment did not increase the number of oocytes retrieved, aligning with Bülow’s findings in that subgroup.

Bülow also reported a systematic review and meta-analysis examining the effects of letrozole cotreatment in COS using gonadotropin preparations other than follitropin delta [20]. The analysis showed that even among normal responders, the number of oocytes retrieved increased by 1.8 oocytes (95% CI: 0.35 to 3.27, p = 0.01) with letrozole cotreatment. While the impact of letrozole on oocyte retrieval remains controversial, this discrepancy may be attributable to the fact that normal responders generally demonstrate sufficient ovarian responses to gonadotropins alone, rendering the additional effect of letrozole minimal and inconsistent across studies. Further high-quality research is needed to determine whether letrozole cotreatment reliably increases the number of oocytes retrieved. However, with regard to follitropin delta, the current dosing algorithm may not appropriately calculate doses for achieving optimal oocyte retrieval in high AMH cases. Our study suggests that letrozole cotreatment could potentially mitigate unexpected poor responses in high AMH patients arising from this limitation.

Two potential mechanisms may explain how letrozole prevents unanticipated suboptimal responses of follitropin delta. The first is the ability of letrozole to enhance endogenous FSH secretion. Letrozole inhibits the activity of the aromatase enzyme, thereby suppressing the conversion of androgens to estradiol and leading to a reduction in serum estradiol levels [13]. This decrease in estradiol acts as a positive feedback signal to the hypothalamus, stimulating the pituitary to increase endogenous FSH secretion [14]. As a result, the combined action of exogenous FSH and endogenous FSH promotes greater follicular development compared to follitropin delta alone, thereby reducing unexpected poor responses. The second mechanism involves the ability of letrozole to enhance follicular sensitivity to FSH. By inhibiting the aromatase enzyme, letrozole reduces the conversion of androgens to estradiol, leading to an increase in intrafollicular androgen concentrations [20,21]. Androgens upregulate the expression of FSH receptors (FSHRs) on follicles, thereby increasing their sensitivity to FSH. This heightened follicular sensitivity likely enhances the response to follitropin delta, thereby reducing unexpected poor responses [22,23]. Previous clinical studies provide support for these mechanisms. For example, Mitwally reported in a prospective pilot study for artificial insemination that letrozole cotreatment reduced the required doses of recombinant or highly purified FSH preparations [15]. Additionally, in COS for IVF/ICSI using gonadotropins other than follitropin delta, letrozole cotreatment was shown to reduce the required gonadotropin dose and shorten the duration of gonadotropin administration [16-18,20]. These findings are consistent with our results.

The exact reasons why COS in patients with high AMH levels exhibits unanticipated suboptimal responses remain unclear. Basic research on polycystic ovary syndrome (PCOS), which is often associated with high AMH levels, has suggested a relationship between FSHR polymorphism and poor follicular development [24]. Another study reported that the SS variant of the FSHR rs6166 polymorphism may be associated with FSH resistance, requiring higher FSH doses for COS [25]. These findings imply that FSHR subtypes or variants observed in patients with high AMH levels might contribute to resistance to follitropin delta. Additionally, there may be a subgroup of high-AMH patients for whom the dose determined by the algorithm is insufficient. To elucidate the causes of the unexpected poor responses to follitropin delta, further basic and clinical research will be essential. However, the hypothesis that the effects of letrozole, enhancing endogenous FSH secretion and increasing FSHR sensitivity, may reduce such unanticipated suboptimal responses appears to be reasonably plausible.

Although the sample size was small, the letrozole cotreatment group did not develop any cases of OHSS in this study. OHSS is a rare but potentially severe complication of COS, with patients having high AMH levels being particularly at risk for its occurrence and severity. Letrozole has been proposed as an effective option for OHSS prevention [26]. Di Guardo and colleagues suggested that letrozole’s ability to reduce estrogen and vascular endothelial growth factor (VEGF) levels may contribute to lowering OHSS incidence [27]. While the efficacy of letrozole in preventing OHSS remains controversial [28], it is generally agreed that letrozole does not exacerbate the risk of OHSS caused by excessive follicular growth [29]. A study on patients at high risk for OHSS has shown that oral administration of letrozole at doses of 2.5 mg, 5.0 mg, and 7.5 mg daily for five days post-oocyte retrieval significantly reduced serum estrogen and VEGF levels. However, while lower doses of 2.5 mg and 5.0 mg showed only marginal trends in reducing OHSS, the higher dose of 7.5 mg significantly reduced its incidence [28]. Tshzmachyan conducted a randomized controlled trial in high-AMH PCOS patients and demonstrated that letrozole co-administration during gonadotropin stimulation significantly reduced OHSS incidence [30]. These findings suggest that letrozole cotreatment during gonadotropin stimulation, as implemented in this study, is beneficial for OHSS prevention. Furthermore, while the dose used in our study was 2.5 mg/day, higher doses may be even more effective in preventing OHSS.

This study is, to the best of our knowledge, the first in the world to demonstrate that letrozole cotreatment can safely prevent unexpected poor responses in COS using follitropin delta. However, several limitations should be acknowledged. First, this was a small-scale study conducted at a single center, which may limit the generalizability of our findings to broader populations and different clinical settings. In particular, the small sample size in the higher follitropin delta dosage groups significantly reduces the statistical power of the results, making it more difficult to draw definitive conclusions. The limited number of patients in these groups increases the risk of Type II errors, potentially leading to an underestimation of true differences or associations. Additionally, the small sample size may contribute to greater variability in outcomes, reducing the reliability and reproducibility of the findings. Second, the retrospective nature of the study inherently introduces potential biases, as data collection and analysis were performed after the treatment outcomes were known. Third, the decision to administer letrozole cotreatment was made at the discretion of the attending physicians rather than being randomized or standardized, which could have led to selection bias. Additionally, due to the observational study design, it was not possible to fully control for confounding factors that may have influenced the observed outcomes. Consequently, the evidence from this study is insufficient to draw definitive conclusions. However, although the study size is small and further investigation is needed, the remarkably high reduction rate of poor response observed in patients with high AMH levels, who are the primary focus of this study, suggests that the clinical utility of letrozole cotreatment is considerable.

Although few in number, there were cases in this study where poor responses persisted despite letrozole cotreatment. The letrozole dose in this study was limited to 2.5 mg/day, reflecting the initial dosage restrictions under the Japanese health insurance system. In contrast, Kar used an initial dose of 5.0 mg/day in their research [23], and other clinical studies, particularly for OHSS prevention, have investigated co-administration at 7.5 mg/day [28]. Further studies are needed to evaluate whether higher doses of letrozole, such as 5.0 mg/day or 7.5 mg/day, could further reduce the risk of poor responses.

Prospective, high-quality research is essential to determine whether letrozole co-treatment should be widely recommended in clinical practice. In parallel, further comprehensive studies are needed to evaluate its impact on key clinical outcomes, including live birth rates, miscarriage rates, the risk of OHSS, and overall patient safety. Additionally, long-term follow-up studies would be beneficial to assess potential effects on maternal and neonatal health, ensuring a thorough understanding of the safety and efficacy of this approach.

## Conclusions

Although high AMH levels typically predict good responses, unexpected poor responses can still occur in COS cycles using follitropin delta. Letrozole cotreatment presents a safe and effective strategy to prevent such unanticipated suboptimal responses. Additionally, it may shorten the duration of ovarian stimulation, reduce the total gonadotropin dosage required, and minimize the risk of OHSS.

These findings have the potential to improve outcomes in ART, particularly for patients who face the challenge of poor responses despite high AMH levels.
